# Diet-derived 25-hydroxyvitamin D3 activates vitamin D receptor target gene expression and suppresses *EGFR* mutant non-small cell lung cancer growth *in vitro* and *in vivo*

**DOI:** 10.18632/oncotarget.6493

**Published:** 2015-12-08

**Authors:** Alissa R. Verone-Boyle, Suzanne Shoemaker, Kristopher Attwood, Carl D. Morrison, Andrew J. Makowski, Sebastiano Battaglia, Pamela A. Hershberger

**Affiliations:** ^1^ Department of Pharmacology and Therapeutics, Roswell Park Cancer Institute, Buffalo, NY 14263, USA; ^2^ Department of Bioinformatics & Biostatistics, Roswell Park Cancer Institute, Buffalo, NY 14263, USA; ^3^ Department of Pathology and Center for Personalized Medicine, Roswell Park Cancer Institute, Buffalo, NY 14263, USA; ^4^ Heartland Assays LLC, Ames, IA 50010, USA

**Keywords:** non-small cell lung cancer (NSCLC), EGFR, vitamin D, CYP27B1, zinc finger nucleases

## Abstract

Epidemiologic studies implicate vitamin D status as a factor that influences growth of *EGFR* mutant lung cancers. However, laboratory based evidence of the biological effect of vitamin D in this disease is lacking. To fill this knowledge gap, we determined vitamin D receptor (VDR) expression in human lung tumors using a tissue microarray constructed of lung cancer cases from never-smokers (where *EGFR* gene mutations are prevalent). Nuclear VDR was detected in 19/19 *EGFR* mutant tumors. Expression tended to be higher in tumors with *EGFR* exon 19 deletions than those with *EGFR* L858R mutations. To study anti-proliferative activity and signaling, *EGFR* mutant lung cancer cells were treated with the circulating metabolite of vitamin D, 25-hydroxyvitamin D3 (25D3). 25D3 inhibited clonogenic growth in a dose-dependent manner. *CYP27B1* encodes the 1α-hydroxylase (1αOHase) that converts 25D3 to the active metabolite, 1,25-dihydroxyvitamin D3 (1,25D3). Studies employing *VDR* siRNA, *CYP27B1* zinc finger nucleases, and pharmacologic inhibitors of the vitamin D pathway indicate that 25D3 regulates gene expression in a VDR-dependent manner but does not strictly require 1αOHase-mediated conversion of 25D3 to 1,25D3. To determine the effects of modulating serum 25D3 levels on growth of *EGFR* mutant lung tumor xenografts, mice were fed diets containing 100 or 10,000 IU vitamin D3/kg. High dietary vitamin D3 intake resulted in elevated serum 25D3 and significant inhibition of tumor growth. No toxic effects of supplementation were observed. These results identify *EGFR* mutant lung cancer as a vitamin D-responsive disease and diet-derived 25D3 as a direct VDR agonist and therapeutic agent.

## INTRODUCTION

Each year in the United States, nearly 24,000 individuals who have never smoked in their lifetime die from lung cancer. This surprisingly places lung cancer in never-smokers among the top 10 deadliest cancers in the United States [1]. A significant fraction of lung cancers that develop in never-smokers contain activating mutations in the Epidermal Growth Factor Receptor (*EGFR*) gene and depend upon EGFR signaling for survival [2, 3]. Large scale sequencing efforts demonstrate that *EGFR* mutations are most frequently detected among lung cancer patients with adenocarcinoma histology, never-smoker status, East Asian ethnicity, and female sex [4]. It is largely unknown why certain demographic populations are at elevated risk for development of *EGFR* mutant lung cancer, but prior pulmonary tuberculosis may be a contributing factor [5]. The identification of additional risk factors has the potential to reveal new approaches to alter disease development or progression.

Emerging pre-clinical and epidemiologic data suggest that low vitamin D levels may favor the growth of *EGFR* mutant lung cancer [6–8]. Vitamin D3 (VD3) is obtained through dietary intake or is synthesized in the skin upon UVB exposure. VD3 is converted within the liver into the primary circulating metabolite, 25-hydroxyvitamin D3 (25D3). Subsequently, 25D3 is converted by the *CYP27B1*-encoded 1α-hydroxylase (1αOHase) into the active metabolite, 1,25-dihydroxyvitamin D3 (1,25D3). 1,25D3 binds to and activates the vitamin D receptor (VDR). Ligand-bound VDR functions as a transcription factor and regulates the expression of genes that control cell differentiation, cell cycle progression, and apoptosis [9–11]. We and others have shown that 1,25D3 inhibits the *in vitro* growth of some but not all lung cancer cell lines [12–14]. Among the cell lines tested by us, HCC827 (*EGFR* del 746–750) and H1975 (*EGFR* L858R/T790M) were found to express relatively high levels of VDR and be particularly sensitive to the growth inhibitory effects of 1,25D3 [14]. We surmised based on our *in vitro* observations that *EGFR* mutant lung cancers are vulnerable to the anti-cancer actions of vitamin D. A corollary to our hypothesis, which is supported by recent epidemiological studies, is that low 25D3 levels increase risk of developing or dying from *EGFR* mutant lung cancer: lower 25D3 levels are associated with a 2.4-fold increased risk of developing *EGFR* mutant adenocarcinoma of the lung [6]. In NHANES III participants, higher serum 25D3 concentrations were associated with decreased risk of dying from lung cancer only among nonsmokers (where *EGFR* gene mutations are more common) [7]. A causal role for 25D3 in controlling growth of *EGFR* mutant lung cancer remains to be established.

High blood 25D3 levels may protect against cancer because as its concentration increases, more 25D3 is locally converted at extra-renal sites by the *CYP27B1*-encoded 1αOHase into the active metabolite, 1,25D3 [15]. Alternatively, 25D3 may act via a *CYP27B1*-independent mechanism of action. When present at high concentrations *in vitro*, 25D3 binds to the VDR and acts as a direct agonist [16, 17]. Furthermore, in *CYP27B1*-null mice that lack any detectable 1,25D3, high dietary vitamin D3 intake (>10,000 IU VD3/kg diet) induces VDR target genes and prevents hypocalcemia and osteomalacia [18]. The contribution of *CYP27B1* to 25D3 activities in *EGFR* mutant lung cancer has not been investigated previously but is important to understand because *CYP27B1* expression is significantly decreased in *EGFR* mutant lung cancer cells and xenografts by erlotinib (supplemental microarray data in [19]), which is used as first-line therapy in patients diagnosed with *EGFR* mutant lung cancer [20]. If 25D3 signals by a *CYP27B1*-independent mechanism, then dietary vitamin D3 supplementation could still be effective when utilized either concomitant with or after erlotinib therapy.

Studies were designed by us to establish the effects of 25D3 on gene regulation and growth of *EGFR* mutant lung cancer *in vitro* and *in vivo*. We also investigated the requirement for *CYP27B1* in mediating the transcriptional effects of 25D3. We identified 25D3 status as a novel host factor that influences the growth of *EGFR* mutant lung cancer and discovered that 25D3 signaling persists despite dramatic reduction in *CYP27B1* expression and 1αOHase activity. The implication of our findings is that dietary vitamin D3 supplementation may be used as a safe and effective approach to increase 25D3 levels and slow the growth of *EGFR* mutant lung cancer, even in tumors that express *CYP27B1* at low levels.

## RESULTS

### *EGFR* mutant NSCLCs express VDR and are therefore targetable by 25D3

**Figure 1:**
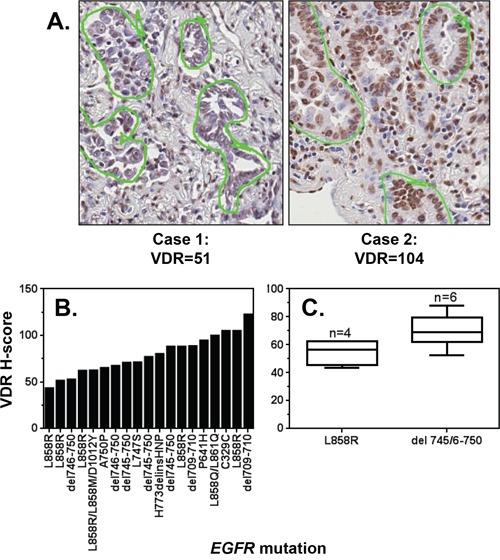
VDR is expressed in *EGFR* mutant tumors A tissue microarray (TMA) was constructed from 84 lung tumors resected from lifetime never-smokers diagnosed with lung cancer. The TMA was stained for VDR, and nuclear expression was quantified by a reviewer blinded to sample identity. **A.** Images of cores in which VDR was detected at relatively low and relatively high levels, respectively. Tumor cells that were scored for VDR are circled in green. **B.** VDR H-scores were calculated for lung tumors that harbor at least one *EGFR* mutation. **C.** The VDR H-scores for cases harboring *EGFR* exon 19 deletions or L858R mutations are displayed. Data are presented only for those cases in which 3 cores were analyzed, and > 100 nuclei per core were scored. The lower, middle, and upper lines of each box represent the lower quartile, median and upper quartile of H-scores.

We postulated based on epidemiologic data that vitamin D signaling can be exploited to suppress the growth of *EGFR* mutant NSCLC. However, the extent to which VDR (the primary component responsible for the biological activity of vitamin D3) is expressed in *EGFR* mutant NSCLC was unknown. To determine if VDR protein is present in *EGFR* mutant NSCLC, we utilized a lung cancer tissue microarray (TMA) constructed within the Pathology Resource Network at Roswell Park Cancer Institute. The array was comprised of 84 lung cancer cases derived from lifetime-never smokers, where *EGFR* mutations are prevalent. VDR protein was detected by immunohistochemistry (Figure 1A). Nuclear VDR expression was quantified using the H-scoring method, which reflects the percentage of cells (0–100) stained at each intensity (0–3). To identify cores that contained *EGFR* gene mutations, targeted sequencing was performed for 48 cases in which matching frozen tissue samples were available for DNA extraction. 19/48 cases (37.5%) had *EGFR* gene mutations (Figure 1B). The common L858R mutation in exon 21 and the exon 19 deletion mutation (del 745/6–750) were detected in 6 and 5 cases, respectively. Compared to patients with exon 21 mutations, lung cancer patients harboring *EGFR* exon 19 deletions have both a better response rate to EGFR TKIs (70–100% vs 20–67% respectively) and improved overall survival (26–34 mos vs 8–17 mos, respectively) [21–23]. Among the samples that contained *EGFR* mutations, all expressed VDR protein (Figure 1B). Cores with *EGFR* Exon 19 deletions tended to have higher VDR H-scores than cases harboring Exon 21 L858R mutations (Figure 1C). However, statistical significance was not achieved, likely due to the small number of cases representing each mutation. Nonetheless, these data demonstrate for the first time that human *EGFR* mutant lung cancers express nuclear VDR protein and may be vulnerable to the anti-cancer activity of vitamin D3.

### 25D3 mediates transcription and growth inhibition in *EGFR* mutant NSCLC cells

**Figure 2:**
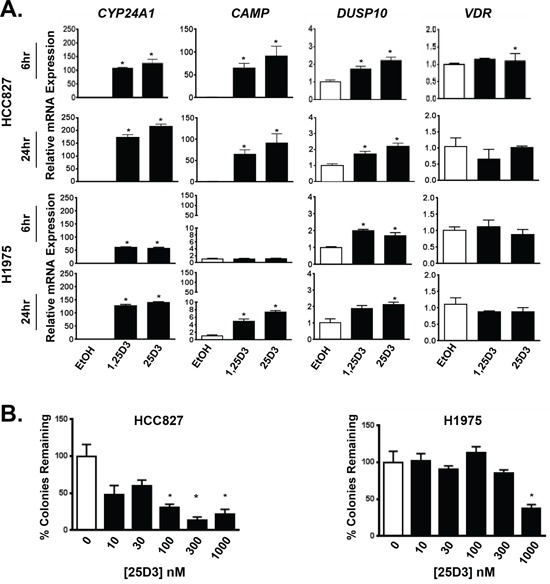
*EGFR* mutant NSCLC cells respond to 25D3 **A.** *EGFR* mutant NSCLC cell lines HCC827 and H1975 were treated with ethanol (EtOH) vehicle control, 100 nM 1,25D3 or 1 μM 25D3. Six and 24 h post treatment, RNA was isolated to study induction of VDR target genes by qRT-PCR. Gene expression was normalized to EtOH controls. **B.** The ability of 25D3 to inhibit clonogenic growth was tested by exposing HCC827 and H1975 cells to 25D3 at increasing concentrations. Data are from triplicate wells within a single experiment and normalized to EtOH-treated controls (0 nM 25D3). Gene expression and clonogenic growth assays were performed a minimum of three times. Error bars represent standard error of the mean. Asterisks indicate *p* < 0.05, treated versus vehicle-control by Student's *t* test.

Given that VDR is present in clinical cases of *EGFR* mutant NSCLC, we next sought to establish the biological activity of 25D3 in *EGFR* mutant NSCLC cells. Two *EGFR* mutant NSCLC cell lines that we previously identified to express relatively high basal levels of VDR were studied [14]: HCC827 cells that harbor an *EGFR* exon 19 deletion mutation (del746–750) and H1975 cells that harbor an L858R mutation and T790M gatekeeper *EGFR* mutation. T790M renders H1975 cells insensitive to EGFR TKI treatment. HCC827 and H1975 cells were treated with either vehicle control, 100 nM 1,25D3 as a positive control, or 1 μM 25D3. RNA was isolated to measure VDR target genes including 24-hydroxylase (*CYP24A1*), cathelicidin antimicrobial peptide (*CAMP*), and dual specificity phosphatase 10 (*DUSP10*) [24–26]. Both vitamin D metabolites induced transcription of all three target genes in both cell lines (Figure 2A). However, stronger gene induction was observed in HCC827 cells. In lung cancer cells, 1,25D3 is well established as an antiproliferative agent [12–14, 27]. To determine if 25D3 also inhibits NSCLC cell growth, clonogenic growth assays were performed. As shown in Figure 2B, clonogenic survival of *EGFR* mutant NSCLC cells was significantly inhibited by 25D3. Greater than 50 percent inhibition was achieved at ≥ 100 nM 25D3 in HCC827 cells and 1 μM 25D3 in H1975 cells. These data demonstrate that 25D3 is active at both the transcriptional and functional levels in *EGFR* mutant NSCLC cells expressing VDR. At disease presentation, the *EGFR* del746–750 mutation (in HCC827 cells) is more commonly found than the T790M mutation (in H1975 cells). Therefore, for the remainder of the studies, we focused on the effects of 25D3 in HCC827 cells.

### 25D3 signaling effects are VDR-dependent

**Figure 3:**
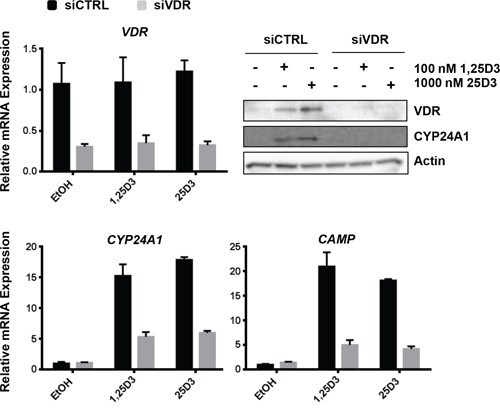
25D3 mediated signaling in *EGFR* mutant, HCC827 cells is dependent upon the VDR HCC827 cells were transfected with either non-targeting control siRNA (siCTRL) or siRNA targeting the VDR (siVDR). Twenty four h later, cells were treated with EtOH vehicle control, 100 nM 1,25D3 or 1 μM 25D3. RNA or protein was extracted 6 h post treatment. qRT-PCR and Western blot assays were performed to study vitamin D regulated genes and to confirm VDR knockdown. The VDR knockdown experiment was repeated 4 times. The results that are presented are from a representative experiment.

25D3 may be converted by lung cancer cells to 1,25D3 [28, 29], which binds with high affinity to VDR to elicit downstream signaling. Alternatively, 25D3 may itself bind VDR to regulate gene expression [17, 18]. To confirm that 25D3 signals through VDR in *EGFR* mutant NSCLC cells, siRNA was used to suppress *VDR* expression. Compared to cells transfected with control siRNA (siCTRL), *VDR* mRNA expression was reduced by 70% in cells transfected with *VDR* siRNA (Figure 3). Cells transfected with siCTRL responded to 25D3 treatment by inducing VDR target gene mRNA and protein expression (Figure 3). In contrast, the transcriptional response to both 1,25D3 and 25D3 was markedly diminished in cells transfected with *VDR* siRNA, and induction of VDR and CYP24A1 protein by vitamin D metabolites was abrogated. We subsequently tested the ability of 25D3 to regulate VDR target gene transcription and inhibit growth in NSCLC cells that endogenously express low levels of VDR. Unlike cells with high VDR expression (such as HCC827, H1975, SK-LU-1, and H292), 25D3 was not effective at inducing target gene transcription or inhibiting colony formation in cells that express relatively low levels of VDR (A427 and A549) (Supplementary Figure S1). The siRNA and cell line studies indicate that the ability of 25D3 to signal in NSCLC cells is VDR dependent.

### Dietary vitamin D3 suppresses tumor growth in HCC827 lung tumor xenografts

**Figure 4:**
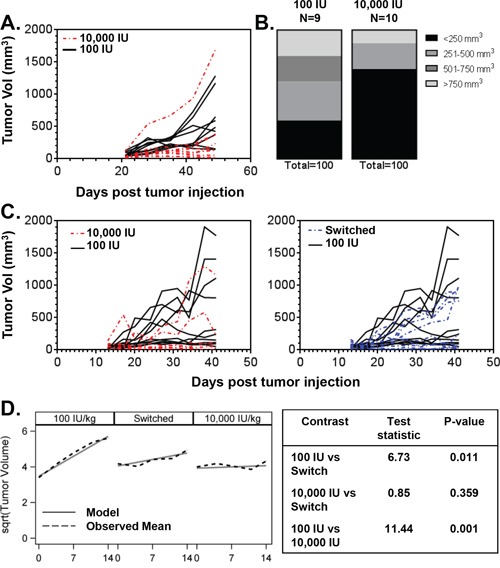
Dietary vitamin D3 supplementation suppresses the growth of *EGFR* mutant lung tumor xenografts **A.** Mice were randomized to receive a diet containing either 100 IU VD3/kg (*n* = 10) or 10,000 IU VD3/kg (*n* = 10). Diets were initiated 7 weeks prior to subcutaneous injection of HCC827 lung cancer cells. Tumor outgrowth was monitored over time using digital calipers. Each line represents the time dependent change in tumor volume within an individual mouse. One mouse in the 100 IU VD3/kg group was removed from the analysis because the implanted tumor cells failed to form a tumor. **B.** Distribution of tumor volumes at end of study for mice that received the 100 IU VD3/kg or 10,000 IUVD3/kg diet. **C.** Mice were fed diets containing either 100 IU VD3/kg (*n* = 20) or 10,000 IU VD3/kg (*n* = 10). Diets were initiated 7 weeks prior to injection of HCC827 cells. One week after tumor cell injection, 10 mice that began on the 100 IU VD3/kg diet were switched to the 10,000 IU VD3/kg diet. All other mice were maintained on their originally assigned research diet. Tumor outgrowth data are presented as described in (A). One mouse in the 10,000 IU VD3/kg group was removed from the analysis because it developed a hematoma. **D.** Tumor growth rates were determined and compared among diet groups for the first 14 d of measured outgrowth, as described in Methods.

Thus far, we have demonstrated that 25D3 is capable of inhibiting the *in vitro* growth of *EGFR* mutant NSCLC cells and inducing VDR target gene expression. Next, we investigated if elevating serum 25D3 concentrations *in vivo* would affect the growth of *EGFR* mutant NSCLC xenografts. To do so, nude mice were placed on research diets containing either 100 international units vitamin D3/kilogram diet (IU VD3/kg) or 10,000 IU VD3/kg diet (*n* = 10 mice per group). Once ingested, VD3 is rapidly converted into 25D3, thereby allowing for a simple method of elevating serum 25D3 concentrations. After seven weeks, when stable 25D3 blood levels were achieved, HCC827 cells were subcutaneously injected into the flank of each mouse. Tumors were first measurable at day 21 post-implantation. At study termination, plasma 25D3 concentrations were 12 ng/mL for mice on the 100 IU VD3/kg diet and 68.5 ng/mL for mice on the 10,000 IU VD3/kg diet. 1,25D3 levels were less variable and averaged 56.3 pg/mL in the 100 IU VD3/kg diet group and 51.4 pg/mL in the 10,000 IU/kg group. The decline in 1,25D3 levels in the 10,000 IU VD3/kg diet group most likely result from suppression of renal *CYP27B1* expression [30]. No toxicity was observed from the VD3-containing diets, as evidenced by normal serum calcium concentrations and steady increase in mouse weights (data not shown). Mice receiving the 10,000 IU VD3/kg diet displayed markedly different tumor outgrowth than mice fed the 100 IU VD3/kg diet (Figure 4A). The tumor volumes of mice receiving the 10,000 IU VD3/kg diet at days 21 (*p* = 0.017), 28 (*p* = 0.043), 35 (*p* = 0.004), and 42 (*p* = 0.010) post-tumor injection were significantly lower than that of mice on the 100 IU VD3/kg diet. At study termination, 70% of mice that received high dietary VD3 and 30% of mice that received low dietary VD3 had tumor volumes < 250 mm^3^ (Figure 4B).

An independent xenograft study was performed to establish whether dietary vitamin D3 supplementation could influence tumor outgrowth when initiated *after* tumor establishment (Figure 4C). Twenty mice were placed on the 100 IU VD3/kg diet, and 10 mice were placed on the 10,000 IU VD3/kg diet (high diet controls). Seven weeks later, HCC827 cells were implanted. One week after tumor implantation, 10 mice that began on the 100 IU VD3/kg diet were switched to the 10,000 IU VD3/kg diet for the remainder of the study. All other mice remained on the research diet to which they had been originally randomized. Pilot studies performed in our laboratory showed that switching diets is an efficient way to rapidly increase serum 25D3 levels: 25D3 increased from 4 ng/mL to 68 ng/mL within one week of switching from the 100 IU VD3/kg diet to the 10,000 IU VD3/kg diet. Tumor measurements began 6 days after diet-switch (day 13 post-tumor injection). Consistent with our first xenograft study, the tumor volumes of mice maintained on the 10,000 IU VD3/kg diet at days 20 (*p* = 0.004), 24 (*p* = 0.004), 27 (*p* = 0.004), 31 (*p* = 0.043), 34 (*p* = 0.026), and 38 (*p* = 0.016) post-injection were significantly lower than that of mice maintained on the 100 IU VD3/kg diet (Figure 4C, left panel). Tumor volumes in mice on the 100 IU VD3/kg diet were not statistically different than mice that were switched to the 10,000 IU VD3/kg diet (Figure 4C, right panel). However, switching from a 100 to 10,000 IU VD3/kg diet was still beneficial, as a significant difference in tumor growth rates between the low and switched diet groups was observed (Figure 4D). Collectively, these data indicate that vitamin D status influences the growth of *EGFR* mutant NSCLC, and that dietary VD3 supplementation may be used to raise serum 25D3 concentration and suppresses *EGFR* mutant NSCLC growth.

### 25D3 signaling persists in *CYP27B1* genetically modified zinc finger nuclease cells

**Figure 5:**
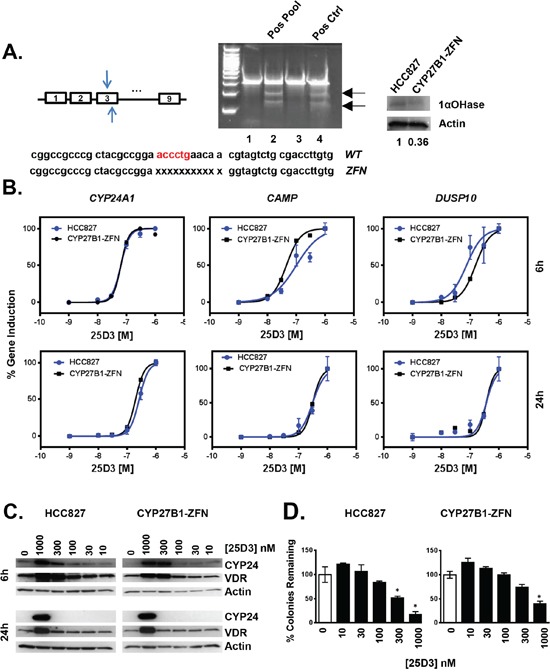
*In vitro* genetic modification of *CYP27B1* using zinc finger nucleases **A.** HCC827 cells were transfected with ZFNs targeting exon 3 of the *CYP27B1* gene. Pools of ZFN transfected cells were tested for genetic modification using the Cel-I assay, as described in Methods. Cel-I generated fragments (indicated with arrows) identify transfected cells in which the *CYP27B1* locus was modified (lane 2, positive pool; lane 4, commercially supplied positive control). When a positive pool was identified, individual clones that comprised the pool were tested for ZFN activity. A positive clone (identified as *CYP27B1*-ZFN) was expanded, sequenced, and found to harbor an eleven base pair deletion in *CYP27B1*. Western blot studies demonstrate that *CYP27B1*-ZFN cells express less 1αOHase protein than parental HCC827 cells. **B.** The ability of 25D3 to induce VDR target gene induction was tested by treating parental and *CYP27B1*-ZFN cells with 25D3 for 6 h or 24 h. RNA was extracted, and expression of *CYP24A1*, *CAMP*, and *DUSP10* was measured by qRT-PCR. **C.** Parental HCC827 and *CYP27B1*-ZFN cells were treated with EtOH vehicle control (0 nM 25D3) or 25D3 at the concentrations indicated for 6 h or 24 h. Protein was extracted, and expression of VDR and CYP241A1 was determined by Western blot. Thirty-five micrograms of protein were analyzed per lane. **D.** The dose-dependent growth inhibitory effects of 25D3 in parental HCC827 and *CYP27B1*-ZFN cells were determined using clonogenic assays, as described in Methods. RNA, protein, and clonogenic data are from one experiment. Each experiment was replicated at least 3 times. Error bars on dose response curves and clonogenic assays represent standard error of the mean for triplicate determinations. Asterisks represent *p* < 0.05 by Student's *t* test, treatment versus control.

*CYP27B1* encodes the 1αOHase that catalyzes conversion of circulating 25D3 into 1,25D3. This conversion occurs within the kidney but also at extra-renal sites including the lung, where *CYP27B1* is expressed [31, 32]. It is widely believed that 25D3 effects on tumor growth are primarily due to its local conversion by 1αOHase to 1,25D3. The results presented above suggest that 25D3 induces VDR target gene transcription, inhibits clonogenic growth, and suppresses outgrowth of *EGFR* mutant lung tumor xenografts. However, it is unclear if the observed effects are due to 25D3 directly or through conversion of 25D3 to 1,25D3. Determining whether 1αOHase is required for 25D3-mediated activity advances the clinical potential of dietary vitamin D3 supplementation because *CYP27B1* expression is significantly decreased in *EGFR* mutant lung cancer cells under pressure of erlotinib treatment both *in vitro* and *in vivo* [19]. To address the requirement for 1αOHase activity, zinc finger nucleases (ZFNs) were used to inactivate the *CYP27B1* gene in HCC827 cells. HCC827 cells were transfected with *CYP27B1* ZFNs, and individual clones were isolated and tested for ZFN activity using the Cel-I mutation detection assay. A ZFN-modified clone was identified, expanded, and found to harbor an 11 base pair deletion at the ZFN cut site (Figure 5A). From the DNA chromatogram, we observed the ZFNs did not result in complete *CYP27B1* inactivation: wild type DNA was still detected in addition to the ZFN-modified sequence. Although over 200 individual clones were screened, we were unable to identify a complete *CYP27B1* knockout. Nonetheless, we hypothesized that alteration of at least one allele may be enough to drastically reduce the 1αOHase activity in HCC827 cells that express *CYP27B1* at relatively low levels (Supplementary Figure S2). Expression of 1αOHase was examined by Western blot. *CYP27B1*-ZFN cells expressed less than half the amount of 1αOHase as parental cells (Figure 5A right panel). We next confirmed that the vitamin D3 signaling axis was intact in the *CYP27B1*-ZFN cells. To do so, cells were treated with 1,25D3, which signals through VDR independent of 1αOHase activity. Compared to parental cells, *CYP27B1*-ZFN cells induced VDR target gene transcription to a similar extent (data not shown). Thus, the ZFN transfection did not adversely affect VDR signaling in HCC827 cells. Next, we tested the ability of 25D3 to induce transcription and growth responses in *CYP27B1*-ZFN cells. Over a range of 25D3 concentrations, parental HCC827 and *CYP27B1*-ZFN cells induced VDR target gene transcription in a comparable manner, as indicated by overlapping dose response curves (Figure 5B). Furthermore, 25D3 induces VDR and CYP24A1 protein expression to a similar extent in parental HCC827 and *CYP27B1*-ZFN cells (Figure 5C). With regard to effects on growth, we achieved statistically significant inhibition in parental HCC827 cells at ≥ 300 nM 25D3. However, 1 μM 25D3 was required to effectively suppresses clonogenic growth in *CYP27B1*-ZFN cells (Figure 5D, right panel). These data indicate that *EGFR* mutant NSCLC cells can tolerate a significant reduction in 1αOHase activity, yet still respond to high dose 25D3 supplementation.

**Figure 6:**
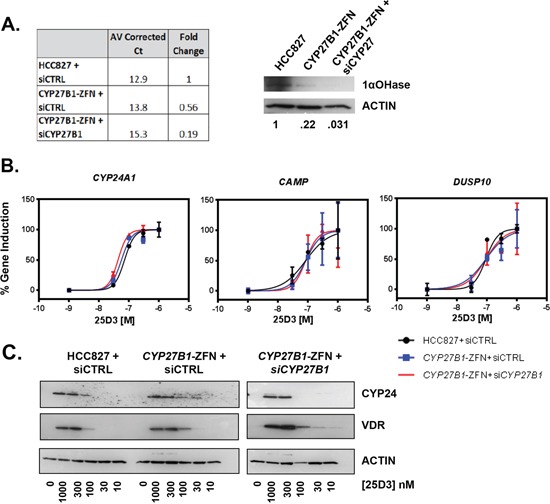
25D3 induces target gene transcription despite marked reduction in *CYP27B1* expression **A.** *CYP27B1* mRNA (left panel) and 1αOHase (right panel) expression was compared 24 h post-transfection of siCTRL into HCC827 and *CYP27B1*-ZFN cells or *CYP27B1* siRNA into *CYP27B1*-ZFN cells. For mRNA expression data, average corrected Ct values were calculated by subtracting the *GAPDH* Ct from the *CYP27B1* Ct value. Fold-change was calculated using the 2-ΔΔCt method. **B.** VDR target gene expression was studied in cells transfected with siRNA for 24 h and then treated with the indicated concentrations of 25D3 for 6 h. qRT-PCR was performed to quantify gene induction. Dose response curves were created in GraphPad Prism. Error bars represent standard error of the mean for triplicate samples within a single experiment. **C.** Induction of VDR and CYP24 protein was examined by Western blot (15 μg total protein per lane). Cells were transfected with siRNA for 24 h and then treated with 25D3 for 6 h. *CYP27B1*-ZFN+ si*CYP27B1* samples were run concurrent with siCTRL-transfected cells but were on a separate gel. All gels were exposed for the same amount of time.

Because we did not achieve a complete *CYP27B1* knockout, *CYP27B1*-specific siRNA was transfected into the ZFN cells to further reduce 1αOHase activity. In *CYP27B1* siRNA transfected cells, *CYP27B1* mRNA levels were reduced 5-fold compared to parental cells and nearly 3-fold compared to ZFN cells (Figure 6A, left panel). 1αOHase expression was lowest in *CYP27B1*-ZFN cells transfected with si*CYP27B1* (Figure 6A right panel). VDR target gene transcription was examined in siRNA transfected cells that were treated with increasing concentrations of 25D3. Despite substantial variation in 1αOHase expression, 25D3 induced VDR target gene transcription to a similar extent in all three cell populations (Figure 6B). Furthermore, VDR and CYP24A1 protein remained induced by 25D3 (Figure 6C). These data suggest that high concentrations of 25D3 surpass the requirement for *CYP27B1* in *EGFR* mutant NSCLC cells.

### 25D3 acts as a VDR agonist when 1αOHase is pharmacologically inhibited, and 1,25D3 production is abolished in *EGFR* mutant NSCLC cells

**Figure 7:**
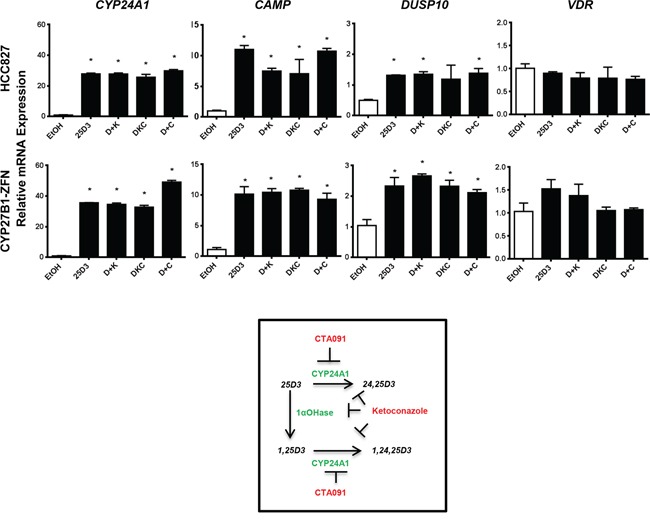
25D3 is active in HCC827 cells in the absence of detectable 1αOHase activity Cells were treated in triplicate with EtOH vehicle control, 1 μM 25D3(D), 1 μM 25D3 + 10 μM Ketoconazole (D+K), 1 μM 25D3 + 50 nM CTA091 (D+C) or 1 μM 25D3 + 10 μM Ketoconazole + 50 nM CTA091 (DKC) for 3 h. RNA was isolated and gene expression quantified by qRT-PCR. The 3 h timepoint was chosen because 1αOHase was 90% inhibited in HCC827 cells and fully inhibited (below the lower limit of quantitation) in *CYP27B1*-ZFN cells. Error bars represent standard error of the mean, and asterisks represent *p* < 0.05 by Student's *t*-test for vehicle versus treated cells.

The above data suggest that 25D3 may serve as a direct VDR agonist when present at high serum concentrations. Because we wanted to test this hypothesis but were unable to achieve a full *CYP27B1* knockout using the ZFNs alone or in combination with siRNA, we took an alternative approach to inhibit 1αOHase pharmacologically with Ketoconazole. Ketoconazole inhibits both 1αOHase and CYP24A1 (IC50 of 28 nM for 1αOHase versus 126 nM for CYP24A1) [33]. HCC827 cells were treated with 25D3, 25D3 plus Ketoconazole, 25D3 plus CTA091, or 25D3 plus Ketoconazole plus CTA091. CTA091 is a selective CYP24A1 inhibitor [34, 35] and is added to prevent CYP24A1-mediated catabolism of 1,25D3. This allows for quantitation of 1,25D3 production in HCC827 cells. To confirm that the inhibitors were functioning properly, we measured the precursor 25D3, the active metabolite 1,25D3, and the CYP24A1-generated catabolite 24,25(OH)2D3 (24,25D3) at 0, 3 and 6 h post-treatment. In parental HCC827 cells, 25D3 was detected at 0 h in all treatment groups, but no additional metabolites were detected. After 3 and 6 h of 25D3 treatment, 24,25D3 and 1,25D3 were detectable (Table 1). When 1,25D3 catabolism was prevented by CTA091, HCC827 cells produced nearly 200 pg/mL of 1,25D3. CTA091 effectively inhibited CYP24A1, as shown by the reduction in 24,25D3 and increase in 1,25D3 production at 3 or 6 h (25D3+CTA091 versus 25D3 alone). 1αOHase inhibition with Ketoconazole was not fully effective, as indicated by residual 1,25D3 production in the 25D3 plus Ketoconazole and 25D3 plus Ketoconazole plus CTA091 treatment groups. *CYP27B1*-ZFN cells were subjected to the same treatments and vitamin D3 metabolite measurements. Compared to parental HCC827 cells, *CYP27B1*-ZFN cells produced significantly less 1,25D3 at 3 and 6 h (Table 1). Additionally, at 3 h, Ketoconazole fully inhibited 1αOHase activity in the ZFN cells: No 1,25D3 production is detected in cells treated with 25D3 plus Ketoconazole or the combination of 25D3, Ketoconazole, and CTA091. At 3 h, when no 1,25D3 production is detected and 1αOHase is fully inhibited in *CYP27B1*-ZFN cells, 25D3 still induces VDR target gene transcription (Figure 7). The most likely explanation for these results is that at pharmacologic levels, 25D3 acts as a direct VDR agonist. Similar trends in gene induction in *CYP27B1*-ZFN cells were observed at 6 h (data not shown).

**Table 1:** Vitamin D3 metabolite production in HCC827 and CYP27B1-ZFN cells

0 hr	25D3 (ng/mL)	24,25D3 (ng/mL)	1,25D3 (pg/mL)	3 hr	25D3 (ng/mL)	24,25D3 (ng/mL)	1,25D3 (pg/mL)	6 hr	25D3 (ng/mL)	24,25D3 (ng/mL)	1,25D3 (pg/mL)
HCC827											
ETOH	<1	<1	<12.5		<1	<1	<12.5		<1	<1	<12.5
25D3	343 (31)	<1	<12.5		271 (12)	33 (0.7)	70.9 (16)		219 (5.6)	59.4 (0.6)	27.9 (3.2)
D+K	312 (9.7)	<1	<12.5		348 (4.7)	3.6 (0.3)	20.5 (5.1)		272 (1.3)	11.9 (0.5)	20.1 (2.2)
DKC	256 (7.1)	<1	<12.5		321 (3.9)	1.2 (0.1)	22.0 (1.3)		264 (5.1)	2.1 (0.2)	19.3 (1.3)
D+C	328 (3.5)	<1	<12.5		338 (21)	1.7 (0.5)	196 (13)		292 (15)	2.9 (0.03)	163 (4.3)
CYP27B1-ZFN											
ETOH	<1	<1	<12.5		<1	<1	<12.5		<1	<1	<12.5
25D3	321 (2.7)	<1	<12.5		281 (7.0)	22.3 (0.6)	53.7 (3.1)		230 (3.3)	58.7 (6.9)	32.2 (1.9)
D+K	301 (12.3)	<1	<12.5		298 (18)	2.2 (0.07)	<12.5		293 (11)	7.6 (0.4)	24.5 (2.3)
DKC	277 (17)	<1	<12.5		283 (5.6)	<1	<12.5		258 (10)	1.5 (0.03)	<12.5
D+C	306 (8.3)	<1	<12.5		308 (31.8)	1.2 (0.08)	101 (5.9)*		288 (5.6)	2.2 (0.09)**	80.6 (0.3)**
Numbers in parenthesis represent standard deviations.											

Cells were treated in duplicate with EtOH vehicle control, 1 μM 25D3(D), 1 μM 25D3 + 10 μM Ketoconazole (D+K), 1 μM 25D3 + 50 nM CTA091 (D+C), or 1 μM 25D3 + 10 μM Ketoconazole + 50 nM CTA091 (DKC) for the times indicated. Cell homogenates were prepared and analyzed for vitamin D3 metabolites by LC-MS/MS. Pairwise comparisons were made between 25D3, 24,25D3 and 1,25D3 levels in HCC827 and *CYP27B1*-ZFN cells treated with D+C. At 3 h post-treatment, 1,25D3 was significantly different between cell lines (*p = 0.01). At 6 h post-treatment, 24,25D3 and 1,25D3 were significantly different between cell lines (**p < 0.01).

## DISCUSSION

Previous work from our laboratory and others identifies 1,25D3 as a potential therapeutic agent in NSCLC [12–14, 36]. Clinical administration of 1,25D3 is hampered by hypercalcemia induction. However, schedules that allow for high, intermittent delivery of 1,25D3 without toxicity have been devised [37, 38]. Unfortunately, using these schedules, no benefit of 1,25D3 administration in unselected patients with advanced NSCLC has been observed [39]. NSCLC is a heterogeneous disease characterized by both histologic variation and the presence of distinct oncogenic driver mutations [40]. We reasoned that beneficial effects of vitamin D could be revealed by (a) focusing on subsets of NSCLC cells that express relatively high levels of VDR protein (*EGFR* mutant NSCLC) and (b) using dietary vitamin D3 to safely and chronically elevate 25D3 levels. When *EGFR* mutant NSCLC cells were exposed to 25D3 *in vitro*, VDR target gene transcription was induced and clonogenic growth was inhibited. Transcriptional and growth responses were shown to be VDR dependent. To test the ability of 25D3 to suppress *EGFR* mutant xenograft growth, we implanted tumor cells in mice that were *pre-fed* diets containing variable levels of VD3. Mice with high dietary VD3 intake (10,000 IU VD3/kg diet) had significantly smaller tumors than mice with low dietary VD3 intake (100 IU VD3/kg diet). Subsequently, we examined the effects of switching mice from the 100 IU VD3/kg diet to the 10,000 IU/kg diet *after* tumors were implanted. Tumor growth rate was significantly reduced in mice that received the diets supplemented with high levels of VD3. These results are the first to identify vitamin D status as a host factor that regulates growth of *EGFR* mutant NSCLC and dietary vitamin D3 supplementation as an effective way to elevate serum 25D3 concentrations and slow tumor growth. Translational potential is supported by our discovery that VDR is expressed in primary human lung tumors that harbor *EGFR* gene mutations.

The ability of 25D3 to control tumor growth has been demonstrated by others. The anti-proliferative actions of 25D3 *in vitro* have been demonstrated in breast, pancreatic and prostate cancer cell lines [41–43]. *In vivo*, dietary vitamin D3 supplementation protects against cancer development [44–49] and inhibits outgrowth of human breast and prostate tumor xenografts [50].

When HCC827 cells were treated with 25D3, we observed potent gene regulation at both 3 h (Figure 7) and 6 h (Figure 2). However, the magnitude of gene induction differed between time points for some (*CYP24A1*, *CAMP*) but not all (*DUSP10*) genes. It has previously been reported that the magnitude of *CYP24A1* mRNA induction by 1,25D3 shows steep time dependence [51]. Matilainen et al demonstrate that 1,25D3 induces a 9-fold increase in *CYP24A1* mRNA within 2 h of treatment but a 400-fold increase within 4 h. Furthermore, subsequent work by the same laboratory demonstrates that not all VDR target genes respond to 1,25D3 treatment with the same kinetics. Rather, the slope of 1,25D3-induced transcription depends upon the basal chromatin accessibility of VDR binding regions [52]. Together, these studies provide a reasonable explanation for why some but not all genes display steep time-dependent differences in vitamin D-mediated gene induction in our model system.

25D3 is metabolized into 1,25D3 by the 1αOHase (encoded by *CYP27B1*). *CYP27B1* is expressed in both histologically normal and lung tumor tissue [31, 32, 53]. However, we (supplemental data) and others [54] find that *CYP27B1* mRNA expression is highly variable across NSCLC cell lines and resected human lung cancers. Variability in *CYP27B1* could restrict the clinical utility of dietary vitamin D3 supplementation. We therefore tested whether 1αOHase-mediated conversion of 25D3 to 1,25D3 was required for signaling in *EGFR* mutant NSCLC cells. To do this, we employed *CYP27B1* specific ZFNs. After screening several hundred clones, we were unable to identify a clone with complete *CYP27B1* inactivation. The clones we tested for ZFN activity were only those that survived limiting dilution cloning and serial passage. We observed that several clones did not survive when we attempted to expand them. It is possible that the non-surviving clones were those with complete loss of *CYP27B1*. However, it is unclear why complete inactivation of *CYP27B1* would result in a lethal phenotype because *CYP27B1* null mice are viable [55–57]. Alternatively, the lack of a *CYP27B1* null clone may be to the inefficiency of the ZFN technology. While several advancements, including heterodimer formation prior to nuclease cutting have been implemented, the ZFNs were reported to have only 5–6% activity when purchased.

Despite our inability to completely inactivate *CYP27B1*, we did successfully abrogate 1αOHase activity by pharmacologically inhibiting residual enzyme in *CYP27B1*-ZFN cells using Ketoconazole (Figure 7, Table 1). In *CYP27B1*-ZFN cells treated with 25D3 plus Ketoconazole plus CTA091, no 1,25D3 production was detected. Yet, 25D3 (1 μM) retained its ability to induce VDR target gene transcription. These observations indicate that at pharmacologic concentrations, 25D3 acts as a VDR agonist in *EGFR* mutant NSCLC. Others have argued for agonist actions of 25D3, and such actions have been detected in the *CYP27B1* null mouse using diets that contain ≥ 10,000 IU VD3/kg [18].

How might our observations be used to inform the use of vitamin D metabolites for management of *EGFR* mutant lung cancers? *CYP27B1*-expressing *EGFR* mutant lung cancer cells (HCC827) are able to produce 1,25D3 from 25D3 (Table 1). In lung cancer patients that harbor tumors of this type, supplementation with physiologic levels of vitamin D3 (as recommended by the Institute of Medicine) would be expected to drive local 1,25D3 production and inhibit tumor growth. Physiologic supplementation might be exploited to prevent disease recurrence among vitamin D-deficient individuals diagnosed with early stage *EGFR* mutant lung cancer who undergo surgical resection with curative intent. In contrast, we surmise that pharmacologic doses of vitamin D3 may be required for therapeutic benefit in individuals diagnosed with advanced *EGFR* mutant lung cancer who receive erlotinib. This is because *CYP27B1* expression decreases significantly under the pressure of erlotinib treatment [19]. An alternative strategy to activate VDR in the context of erlotinib-driven *CYP27B1* loss would be to systemically administer the active metabolite, 1,25D3. Supporting the viability of this approach, we find that pharmacologic concentrations of 1,25D3 (100 nM) induce gene expression and inhibit the growth of *CYP27B1*-ZFN cells (data not shown). Further studies using *CYP27B1*-ZFN xenografts will be required to determine whether oral vitamin D3 supplementation and weekly systemic administration of 1,25D3 provide comparable therapeutic benefit and safety *in vivo*.

Prior to our studies, the expression of VDR in primary human *EGFR* mutant lung tumors was unknown. To fill this gap in knowledge, we analyzed nuclear VDR expression in tumor cores from lifetime never-smokers that had corresponding DNA available for targeted sequencing of the *EGFR* gene. We focused on never-smokers because *EGFR* mutations are more commonly observed in this subset of patients. Among the 19 cases of *EGFR* mutant lung cancer that were identified, we observed variation in VDR expression and cell morphology. Of note, tumors that harbored *EGFR* exon 19 deletions tended to have higher VDR expression than those that harbored *EGFR* L858R mutations. This is intriguing because patients with *EGFR* exon 19 deletions have better response rates to erlotinib and overall survival compared to patients with *EGFR* L858R mutations [21–23].

We also observed in our TMA studies that tumor cell morphology in cores with higher VDR expression appears more epithelial-like. This may suggest that vitamin D signaling plays a role in maintaining tumor cells in an epithelial state. Such an observation is consistent with our prior which shows that 1,25D3 induces expression of the epithelial cell marker E-cadherin and opposes the EMT-promoting actions of TGF-β in lung cancer cells [58]. EMT initiating events including β-catenin nuclear translocation have recently been shown to be essential for the growth of *EGFR* mutant lung cancers [36]. A vitamin D3-induced increase in E-cadherin expression and localization at the plasma membrane could influence β-catenin localization and prohibit its nuclear actions. EMT is also a mechanism by which *EGFR* mutant lung cancer cells become resistant to erlotinib therapy [19, 59–61]. Potentially, vitamin D-mediated maintenance of epithelial phenotype may delay EMT onset and render *EGFR* mutant NSCLC cells responsive to EGFR TKI therapy for an extended period of time. The ability of high dose vitamin D3 supplementation to delay erlotinib resistance is currently under investigation in our laboratory.

## MATERIALS & METHODS

### Cell culture

All cell lines were purchased from American Type Culture Collection (ATCC, Manassas, VA, USA). HCC827 and H1975 cells were cultured in RPMI 1640 supplemented with 10% fetal bovine serum (FBS) (Tissue Culture Biologicals, Tulare, CA, USA) and 1% penicillin-streptomycin. H292 cells were cultured in the same media, but the media was also supplemented with 2 mM glutamine, 1 mM sodium pyruvate, and 10 mM HEPES buffer. SK-LU-1 and A427 cells were maintained in EMEM media supplemented with 10% FBS and antibiotics, and A549 cells were cultured in BME media with FBS, antibiotics, and 2 mM glutamine (Corning, Tewksbury, MA, USA). Cells were maintained at 37°C and 5% CO2. All cell lines tested negatively for mycoplasma. HCC827 cells, the primary cell line used in these studies, were authenticated by RADIL.

### Reagents and chemicals

A 480 μM stock of 1,25D3 in 100% ethanol was kindly provided by Dr. Candace Johnson (Roswell Park Cancer Institute, Buffalo, NY, USA). For all treatments, 1,25D3 was diluted in cell culture media to a final concentration of 100 nM. The vitamin D metabolite, 25D3, was purchased from BioMol and prepared as a 1 mg/mL (2.5 mM) stock in 100% ethanol. Prior to use, 25D3 was diluted in cell culture media to a final concentration of 1 μM. Ketoconazole was purchased from Sigma Aldrich, resuspended in DMSO and used at final concentrations of 10 μM. The CYP24A1 inhibitor, CTA091, was kindly provided by Martin Petkovich (martin.petkovich@queensu.ca) and OPKO Health (Miami, FL) and was diluted in cell culture media to a final concentration of 50 nM on the day of use.

### RNA isolation and cDNA synthesis

Cells were seeded at a density of 2 × 10^5^ cells per well of a six well plate. For time-course gene expression studies, cells were seeded and treated in charcoal stripped serum (CSS) with antibiotics (Figure 2, Figure 7). All other gene expression studies were performed in serum free media supplemented with antibiotics (Figures 5, 6). Cells were collected in TRI-reagent (Direct-Zol, RNA Mini-Prep Kit, Zymo Research, Irvine, CA, USA) to extract RNA. Isolation was carried out following the manufacturer's instructions. A NanoDrop was used to measure RNA concentrations. All RNA had a 260/280 ratio of at least 1.98. RNA was stored at −80°C. 500 ng of RNA was reverse transcribed into cDNA in a final volume of 20 μL using the High Capacity cDNA Reverse Transcription Kit with the addition of an RNAse inhibitor (Applied Biosystems, Foster City, CA, USA), according to the manufacturer's instructions.

### Real-time PCR

The SYBR green/ROX/qPCR Master Mix kit (Thermo Fisher Scientific, Pittsburgh, PA, USA) was utilized for qRT-PCR reactions. PCR was performed using a 7300 Real Time PCR System (Applied Biosystems). cDNA was used at a volume of 1 μL per reaction. Reactions were run at 50°C for 2 min, 95°C for 10 min, then 40 cycles of 95°C for 15 sec and 60°C for one min. Data was collected during the 60°C extension step. The ΔΔCt method was used for calculating relative gene expression levels. Primers were purchased from Integrated DNA Technologies and sequences are as follows:

**Table:** 

***CYP24A1***	F: 5′ GCA CAA GAG CCT CAA CAC CAA 3′
	R: 5′ AGA CTG TTT GCT GTC GTT TCC A 3′
***VDR***	F: 5′ ATA AGA CCT ACG ACC CCA CCT A 3′
	R:GGA CGA GTC CAT CAT GTC TGA A 3′
***CYP27B1***	F: 5′ CAG TCC ATC CTG GGA AAT GTG A 3′
	R: 5′ ACC ACA GGG TAC AGT CTT AGC A 3′
***DUSP10***	F: 5′ TTG AGT TCA TTG AGG AAG CTC ACC 3′
	R: 5′ AAC TCT AGC AAC TGC CCC ATG AAG 3′
***CAMP***	F: 5′TTC AAG AAG GAC GGG CTG GTG AAG 3′
	R: 5′ GTC CTG GGT ACA AGA TTC CGC AAA 3′
***GAPDH***	F: 5′ CTC CTC TGA CTT CAA CAG CG 3′
	R: 5′GCC AAA TTC GTT GTC ATA CCA G 3′

### Clonogenic growth assays

Cells were seeded in triplicate at a low density optimized for each cell line (HCC827–650 cells/well, H1975–650 cells/well, SK-LU-1–700 cells/well, H292–650 cells/well, and A549–650 cells/well). Cells were seeded and treated in complete growth medium. Treatments included vehicle control, 1,25D3, or 25D3 and were repeated every 2 days for 10–14 days. Colonies were fixed by adding 70% methanol per well for 5 min. This fixation step was repeated, followed by staining with 0.1% crystal violet for 5 min. Water was used to rinse the wells. Plates were dried at least 24 h prior to counting colonies. Colonies were inspected using a microscope, and a colony was defined as a cluster containing at least 30 cells. The calculation for percent colonies remaining for each treatment was calculated as follows:

$$\begin{array}{c} \text{\% Colonies}\\ \text{Remaining} \end{array}\}=100\%*\frac{\text{Number of colonies for treatment group}}{\text{Average number of colonies for treatment group}}$$

### siRNA transfections

Cells were seeded in triplicate at a density of 2 × 10^5^ cells per well in antibiotic free media. siRNA targeting the *VDR* (siGenome, Thermo Fisher Scientific, cat# L-003448–00), *CYP27B1* (Santa Cruz, Dallas TX), or the respective controls (On-target Plus Control Pool, cat#D001810–10-05, or Control siRNA-A, cat# sc-37007), were transfected into cells in a solution containing Dharmafect reagent 1 (Dharmacon RNA Technologies, Lafayette CO) and Opti-MEM (Thermo Fisher Scientific Grand Island NY). The final concentration of siRNAs was 10 nM. The next day, cells were washed with serum free media containing 1% antibiotics. Twenty-four h post transfection, cells were treated with vehicle control, 1,25D3 or 25D3. Cells were harvested 6 h later for RNA and protein analysis.

### Protein extraction and western blot analysis

Cells for protein analysis were collected into tissue culture media and centrifuged at 118 × *g* for 6 min. Media was aspirated and the cell pellet was resuspended and washed in ice cold PBS. The wash was removed, and proteins were extracted in Triton-X100/SDS lysis buffer as previously described [62]. Cell pellets that were not analyzed immediately were stored at −80°C. VDR (Thermo Scientific, clone MA1–710) and CYP24A1 (Sigma, Clone 1F8) were detected by Western blot, as previously described [62]. Antibodies specific for 1αOHase were obtained from ThermoFisher and The Binding Site.

### Vitamin D metabolite measurements via LC-MS/MS

Cells (1 × 10^6^) were seeded into T25 flasks and treated with either vehicle control, 1 μM 25D3, 10 μM Ketoconazole, 1 μM 25D3 plus 10 μM Ketoconazole, 1 μM 25D3 plus 10 μM Ketoconazole plus 50 nM CTA091, or 50 nM CTA091 plus 1 μM 25D3. All treatments were done in tissue culture media containing 10% charcoal-stripped serum. At designated times, cells were scraped up, and the cell/media suspension was flash frozen in liquid nitrogen. Samples were shipped frozen to Heartland Assays for determination of 1,25D3, 25D3 and 24,25D3 levels by LC-MS/MS.

25D3 and 24,25D3 were extracted using a liquid-liquid-extraction protocol. The procedure was the same for both analytes, except the sample volumes were doubled for 24,25D3 to increase the concentration of metabolite. Experimental samples, calibration curve, and NIST controls were pipetted into boro-silicate test tubes and then spiked with deuterated internal standard of each metabolite being measured (deuterated and non-deuterated 25D3 neat reagents were purchased from Sigma-Aldrich). Then ZnSO4 [63] and methanol were added to the samples to precipitate proteins. Hexanes were then added and the samples vortexed. The samples were centrifuged, and the organic hexane layer was collected and dried using a savant dryer. The samples were re-constituted into LCMS grade methanol and water both with 0.1% formic acid, injected onto an Agilent 1290 HPLC with an Agilent Poroshell 120 PFP 2.7 um 2.1 × 100 mm column coupled to an Agilent 6460 Triple-quad mass spectrometer with electrospray ionization source (ESI) in positive mode, and then analyzed using Masshunter software. The 25D3 assay was run with NIST certified serum standards. The extraction efficiency of 25D3 was 97%. The 24,25D assay utilized a deuterated internal standard (d6–24,25D3 from Medical Isotopes Inc. and non-deuterated 24,25D2 and 24,25D3 from Sigma-Aldrich). The extraction efficiency of 24,25D3 was 96%, based on spiked sample recovery.

For analysis of 1,25D3, experimental samples, standard curve, and quality control (QC) samples were pipetted into boro-silicate test tubes and then spiked with d6–1,25D3 (Sigma-Aldrich). Proteins were precipitated with equal parts acetonitrile to sample/standards/QC. After acetonitrile addition, the samples were centrifuged and poured into equal parts water. The samples were extracted via solid phase extraction (SPE) using Diasorin C18-OH 0.5 g columns on a VAC-ELUT apparatus. After SPE, the eluate was dried down and derivatized using 0.75 mg/mL PTAD (Sigma-Aldrich) in acetonitrile for 2 h at room temperature [64] The reactions were quenched with water, vortexed, and dried down. The samples were reconstituted into LCMS grade methanol and water both with 0.1% formic acid, and then injected onto an Agilent 1290 HPLC with an Agilent ZORBAX RRHD C18 1.8 um 2.1 × 50 mm column coupled to an Agilent 6460 Triple-quad mass spectrometer with ESI in positive mode and analyzed using Masshunter software. 1,25D3 extraction efficiency was 81% based on lyophilized, certified DiaSorin standard that was reconstituted with charcoal-stripped DiaSorin 0 calibrator serum.

### *CYP27B1* zinc finger nucleases and Cel-I mutation detection assay

Custom CompoZr zinc finger nucleases (ZFN) targeting *CYP27B1* were purchased from Sigma Aldrich. A ZFN mRNA pair was transfected into cells seeded at a density of 4 × 10^5^ cells per well in antibiotic free medium. The final amount of zinc finger mRNA added per transfection was 2 μg. TransIT and Boost (Mirus) transfection reagents were used at a volume of 1 μL per well of a 6-well plate. Medium was changed on transfected cells the following morning. Forty-eight hours later, cells were partitioned into three aliquots to allow for genomic DNA extraction, limiting dilution cloning, and maintenance/propagation. Genomic DNA extraction was performed using the ChargeSwitch Kit from Invitrogen. *CYP27B1* primers specific for exon 3 were used to amplify genomic DNA via PCR. A portion of the PCR sample was saved for detection of *CYP27B1* products on a 1.2% agarose gel. To conduct the CEL-I mutation detection assay, the 20 μL PCR product was denatured/renatured by running the following program on a thermocycler: 95°C 10 min, 95°C to 85°C, −2°/second, 85°C to 25°C, −1°C/second, 4°C indefinitely. To this sample, 2μL of nuclease S, 2μL of enhancer, and 2.4 μL of MgCl2 were added (Surveyor Mutation Detection Kit, Transgenomic). The reaction was incubated at 42°C for 60 minutes and then terminated by adding 2.6 μL of Stop solution. 6X sample buffer was added and the product was run on a 10% PAGE-TBE gel (BioRad) in 1X TBE Buffer for 90 minutes at 100 V. The gel was then exposed to a solution of 1X TBE and Ethidium Bromide for 5 minutes and imaged using a Gel Doc (BioRad, Quantity One Image Analysis). ZFN modified DNA was indicated by the presence of specific Cel-I generated restriction fragments. The positive clone that demonstrated the greatest cutting in the Cel-I assay was expanded and denoted as *CYP27B1*-ZFN. Exon 3 of the *CYP27B1* gene was sequenced in the Roswell Park Cancer Institute Genomics Core and found to contain an 11 base pair deletion at the targeted ZFN cut site. Cel-I assays were performed after multiple passages of *CYP27B1*-ZFN cells to confirm maintenance of the ZFN-modified *CYP27B1* alleles.

### HCC827 xenograft studies

Xenograft studies were performed under an IACUC approved protocol, within the AAALAC-certified laboratory animal resource at Roswell Park Cancer Institute. For Study 1: 20 female nude mice between 4–6 weeks of age were purchased from Harlan Laboratories. Immediately upon receipt, mice were randomized to receive diets containing either 100 IU VD3/kg (*n* = 10) or 10,000 IU VD3/kg (*n* = 10) (Research Diets Inc, New Brunswick NJ). All other components of the diet were identical. After 7 weeks on the diets, mice were subcutaneously injected in the rear flank with HCC827 cells (5 × 10^6^) in 200 μL PBS. Once tumors were palpable, tumor measurements and mouse weights were obtained once weekly. Tumor volumes were calculated using the equation: Volume = (L × W^2^)/2. When at least one tumor reached 1500 mm^3^, the study was terminated. Blood was collected into heparinized syringes for determination of 25D3 (University of Pittsburgh) and 1,25D3 (Heartland Assays). Graphs of tumor outgrowth data were created using GraphPad Prism 6.

For Study 2: 30 female nude mice between 4–6 weeks of age were purchased from Harlan Laboratories. Upon arrival, mice were randomized to receive research diets containing either 100 IU VD3/kg (*n* = 20) or 10,000 IU VD3/kg (*n* = 10). Seven weeks later, 5 × 10^6^ HCC827 cells in PBS were subcutaneously injected into the flank of each mouse. One week after tumor implantation, 10 mice that began on the 100 IU VD3/kg diet were switched to the 10,000 IU/kg diet. All other mice were maintained on the diets to which they were originally assigned. Measurable tumors were present on day 13 post-injection. Tumor volumes were measured twice weekly using digital calipers, and animal weights were measured once weekly thereafter. The study was terminated when at least one tumor reached a volume > 1500 mm^3^ (confirmed by two independent measurements at least 4 d apart).

Statistical analysis was performed in collaboration with the Roswell Park Cancer Institute Bioinformatics and Biostatistics Department. Median tumor volumes were log-transformed and compared using a Wilcoxon rank sum test. In Study 2, tumor growth rates were calculated by modeling the data (for first 14 days of measured tumor outgrowth) using a linear mixed model. Tumor volumes were transformed and modeled as a function of time, diet, their interaction and a nested random mouse effect. A first order auto-regressive function was used for the covariance matrix. Model assumptions were graphically verified using residual plots and quantile-quantile plots. The resultant data presented the need to implement a ¼ root transformation.

### Tissue microarray

A tissue microarray consisting of tumors resected from individuals who were lifetime-never smokers and diagnosed with lung cancer was developed through the Pathology Resource Network (PRN) at Roswell Park Cancer Institute. The TMA was comprised of 84 patients, with 3 tissue cores arrayed per patient. The TMA was stained for VDR (Affinity Bio.; # MAI-710) as described previously [65] and scored by a researcher blinded to sample identity using Aperio software. Only VDR staining in tumor cell nuclei was scored. The number of nuclei scored per case was variable and depended upon the size of cores and the density of tumor within each core (range 241–1348). Cores were scored by assigning the percent of positively stained nuclei a value between 0–3, with 0 representing no staining and 3 representing intense staining. H-scores were calculated for each core, as follows:

H Score=(3*% nuclei stained at 3)+(2*% nuclei stained at 2)+(1*% nuclei stained at 1)

DNA from histologically normal lung tissue and lung tumor tissue was available for 48 of the cases included in the TMA and was used for targeted DNA sequencing (Agilent HaloPlex) to study the *EGFR* mutational landscape. Sequencing was performed within the Genomics Core Facility at Roswell Park Cancer Institute.

### Statistical analysis for *in vitro* studies

All comparisons of gene expression and clonogenic growth (treatment versus vehicle controls) were performed using an unpaired *t*-test in GraphPad Prism 6, where asterisks represent *p* < 0.05.

## SUPPLEMENTARY FIGURES



## References

[1] Samet JM, Avila-Tang E, Boffetta P, Hannan LM, Olivo-Marston S, Thun MJ, Rudin CM (2009). Lung cancer in never smokers: clinical epidemiology and environmental risk factors. Clinical Cancer Research.

[2] Pao W, Miller V, Zakowski M, Doherty J, Politi K, Sarkaria I, Singh B, Heelan R, Rusch V, Fulton L, Mardis E, Kupfer D, Wilson R, Kris M, Varmus H (2004). EGF receptor gene mutations are common in lung cancers from “never smokers” and are associated with sensitivity of tumors to gefitinib and erlotinib. Proceedings of the National Academy of Sciences of the United States of America.

[3] Toyooka S, Tokumo M, Shigematsu H, Matsuo K, Asano H, Tomii K, Ichihara S, Suzuki M, Aoe M, Date H, Gazdar AF, Shimizu N (2006). Mutational and epigenetic evidence for independent pathways for lung adenocarcinomas arising in smokers and never smokers. Cancer Res.

[4] Rosell R, Moran T, Queralt C, Porta R, Cardenal F, Camps C, Majem M, Lopez-Vivanco G, Isla D, Provencio M, Insa A, Massuti B, Gonzalez-Larriba JL, Paz-Ares L, Bover I, Garcia-Campelo R (2009). Screening for epidermal growth factor receptor mutations in lung cancer. The New England Journal of Medicine.

[5] Luo YH, Wu CH, Wu WS, Huang CY, Su WJ, Tsai CM, Lee YC, Perng RP, Chen YM (2012). Association between tumor epidermal growth factor receptor mutation and pulmonary tuberculosis in patients with adenocarcinoma of the lungs. Journal of Thoracic Oncology.

[6] Shin DY, Kim S, Park S, Koh JS, Kim CH, Baek H, Yang SH, Na II (2014). Serum 25-hydroxyvitamin D levels correlate with EGFR mutational status in pulmonary adenocarcinoma. Endocrine-Related Cancer.

[7] Cheng TY, Neuhouser ML (2012). Serum 25-hydroxyvitamin D, vitamin A, and lung cancer mortality in the US population: a potential nutrient-nutrient interaction. Cancer Causes & Control : CCC.

[8] Cheng TY, Lacroix AZ, Beresford SA, Goodman GE, Thornquist MD, Zheng Y, Chlebowski RT, Ho GY, Neuhouser ML (2013). Vitamin D intake and lung cancer risk in the Women's Health Initiative. The American Journal of Clinical Nutrition.

[9] Swami S, Raghavachari N, Muller UR, Bao YP, Feldman D (2003). Vitamin D growth inhibition of breast cancer cells: gene expression patterns assessed by cDNA microarray. Breast Cancer Research and Treatment.

[10] Krishnan AV, Shinghal R, Raghavachari N, Brooks JD, Peehl DM, Feldman D (2004). Analysis of Vitamin D-Regulated Genes in LNCaP Human Prostate Cancer Cells Using cDNA Microarrays. The Prostate.

[11] White JH (2004). Profiling 1,25-dihydroxyvitamin D3-regulated gene expression by microarray analysis. Steroid Biochemistry and Molecular Biology.

[12] Jeong Y, Xie Y, Lee W, Bookout AL, Girard L, Raso G, Behrens C, Wistuba II, Gadzar AF, Minna JD, Mangelsdorf DJ (2012). Research resource: Diagnostic and therapeutic potential of nuclear receptor expression in lung cancer. Molecular Endocrinol.

[13] Kim SH, Chen G, King AN, Jeon CK, Christensen PJ, Zhao L, Simpson RU, Thomas DG, Giordano TJ, Brenner DE, Hollis B, Beer DG, Ramnath N (2012). Characterization of vitamin D receptor (VDR) in lung adenocarcinoma. Lung Cancer.

[14] Zhang Q, Kanterewicz B, Shoemaker S, Hu Q, Liu S, Atwood K, Hershberger P (2013). Differential response to 1alpha,25-dihydroxyvitamin D3 (1alpha,25(OH)2D3) in non-small cell lung cancer cells with distinct oncogene mutations. The Journal of Steroid Biochemistry and Molecular Biology.

[15] Feldman D, Krishnan AV, Swami S, Giovannucci E, Feldman BJ (2014). The role of vitamin D in reducing cancer risk and progression. Nat Rev Cancer.

[16] Hughes MR, Baylink DJ, Jones PG, Haussler MR (1976). Radioligand receptor assay for 25-hydroxyvitamin D2/D3 and 1 alpha, 25-dihydroxyvitamin D2/D3. J Clin Invest.

[17] Lou YR, Molnar F, Perakyla M, Qiao S, Kalueff AV, St-Arnaud R, Carlberg C, Tuohimaa P (2010). 25-Hydroxyvitamin D(3) is an agonistic vitamin D receptor ligand. The Journal of Steroid Biochemistry and Molecular Biology.

[18] Rowling MJ, Gliniak C, Welsh J, Fleet JC (2007). High dietary vitamin D prevents hypocalcemia and osteomalacia in CYP27B1 knockout mice. The Journal of Nutrition.

[19] Zhang Z, Lee JC, Lin L, Olivas V, Au V, LaFramboise T, Abdel-Rahman M, Wang X, Levine AD, Rho JK, Choi YJ, Choi CM, Kim SW, Jang SJ, Park YS, Kim WS (2012). Activation of the AXL kinase causes resistance to EGFR-targeted therapy in lung cancer. Nature Genetics.

[20] Zhou C, Wu YL, Chen G, Feng J, Liu XQ, Wang C, Zhang S, Wang J, Zhou S, Ren S, Lu S, Zhang L, Hu C, Hu C, Luo Y, Chen L (2011). Erlotinib versus chemotherapy as first-line treatment for patients with advanced EGFR mutation-positive non-small-cell lung cancer (OPTIMAL, CTONG-0802): a multicentre, open-label, randomised, phase 3 study. The Lancet Oncology.

[21] Won YW, Han JY, Lee GK, Park SY, Lim KY, Yoon KA, Yun T, Kim HT, Lee JS (2011). Comparison of clinical outcome of patients with non-small-cell lung cancer harbouring epidermal growth factor receptor exon 19 or exon 21 mutations. Journal of Clinical Pathology.

[22] Zhang Y, Sheng J, Kang S, Fang W, Yan Y, Hu Z, Hong S, Wu X, Qin T, Liang W, Zhang L (2014). Patients with exon 19 deletion were associated with longer progression-free survival compared to those with L858R mutation after first-line EGFR-TKIs for advanced non-small cell lung cancer: a meta-analysis. PloS One.

[23] Lim SH, Lee JY, Sun JM, Ahn JS, Park K, Ahn MJ (2014). Comparison of clinical outcomes following gefitinib and erlotinib treatment in non-small-cell lung cancer patients harboring an epidermal growth factor receptor mutation in either exon 19 or 21. Journal of Thoracic Oncology.

[24] Chen K-S, Prahl JM, DeLuca HF (1993). Isolation and expression of human 1,25-dihydroxyvitamin D3 24-hydroxylase cDNA. Proc Natl Acad Sci USA.

[25] Peehl DM, Shinghal R, Nonn L, Seto E, Krishnan AV, Brooks JD, Feldman D (2004). Molecular activity of 1,25-dihydroxyvitamin D3 in primary cultures of human prostatic epithelial cells revealed by cDNA microarray analysis. The Journal of Steroid Biochemistry and Molecular Biology.

[26] Gombart AF, Borregaard N, Koeffler HP (2005). Human cathelicidin antimicrobial peptide (CAMP) gene is a direct target of the vitamin D receptor and is strongly up-regulated in myeloid cells by 1,25-dihydroxyvitamin D3. FASEB Journal.

[27] Higashimoto Y, Ohata M, Nishio K, Iwamoto Y, Fujimoto H, Uetani K, Suruda T, Nakamura Y, Funasako M, Saijo N (1996). 1 alpha, 25-dihydroxyvitamin D3 and all-trans-retinoic acid inhibit the growth of a lung cancer cell line. Anticancer Research.

[28] Mawer EB, Hayes ME, Heys SE, Davies M, White A, Stewart MF, Smith GN (1994). Constitutive synthesis of 1,25-dihydroxyvitamin D3 by a human small cell lung cancer cell line. The Journal of Clinical Endocrinology and Metabolism.

[29] Jones G, Ramshaw H, Zhang A, Cook R, Byford V, White J, Petkovich M (1999). Expression and activity of Vitamin D-Metabolizing Cytochrome P450s (CYP1a and CYP24) in Human Nonsmall Cell Lung Carcinomas. Endocrinology.

[30] Fleet JC, Gliniak C, Zhang Z, Xue Y, Smith KB, McCreedy R, Adedokun SA (2008). Serum metabolite profiles and target tissue gene expression define the effect of cholecalciferol intake on calcium metabolism in rats and mice. The Journal of Nutrition.

[31] Anderson PH, Hendrix I, Sawyer RK, Zarrinkalam R, Manavis J, Sarvestani GT, May BK, Morris HA (2008). Co-expression of CYP27B1 enzyme with the 1. 5kb CYP27B1 promoter-luciferase transgene in the mouse. Molecular and Cellular Endocrinology.

[32] Hansdottir S, Monick MM, Hinde SL, Lovan N, Look DC, Hunninghake GW (2008). Respiratory epithelial cells convert inactive vitamin D to its active form: potential effects on host defense. J Immunol.

[33] Schuster I, Egger H, Bikle D, Herzig G, Reddy GS, Stuetz A, Stuetz P, Vorisek G (2001). Selective inhibition of vitamin D hydroxylases in human keratinocytes. Steroids.

[34] Kahraman M, Sinishtaj S, Dolan PM, Kensler TW, Peleg S, Saha U, Chuang SS, Bernstein G, Korczak B, Posner GH (2004). Potent, selective and low-calcemic inhibitors of CYP24 hydroxylase: 24-sulfoximine analogues of the hormone 1alpha,25-dihydroxyvitamin D(3). J Med Chem.

[35] Posner GH, Helvig C, Cuerrier D, Collop D, Kharebov A, Ryder K, Epps T, Petkovich M (2010). Vitamin D analogues targeting CYP24 in chronic kidney disease. The Journal of Steroid Biochemistry and Molecular Biology.

[36] Nakagawa K, Kawaura A, Kato S, Takeda E, Okano T (2005). 1{alpha},25-Dihydroxyvitamin D3 is a preventive factor in the metastasis of lung cancer. Carcinogenesis.

[37] Beer TM, Munar M, Henner W.D (2001). A Phase I Trial of Pulse Calcitriol in Patients with Refractory Malignancies. Cancer.

[38] Fakih MG, Trump DL, Muindi JR, Black JD, Bernardi RJ, Creaven PJ, Schwartz J, Brattain MG, Hutson A, French R, Johnson CS (2007). A phase I pharmacokinetic and pharmacodynamic study of intravenous calcitriol in combination with oral gefitinib in patients with advanced solid tumors. Clinical Cancer Research.

[39] Ramnath N, Daignault-Newton S, Dy GK, Muindi JR, Adjei A, Elingrod VL, Kalemkerian GP, Cease KB, Stella PJ, Brenner DE, Troeschel S, Johnson CS, Trump DL (2013). A phase I/II pharmacokinetic and pharmacogenomic study of calcitriol in combination with cisplatin and docetaxel in advanced non-small-cell lung cancer. Cancer Chemotherapy and Pharmacology.

[40] Sholl LM, Aisner DL, Varella-Garcia M, Berry LD, Dias-Santagata D, Wistuba II, Chen H, Fujimoto J, Kugler K, Franklin WA, Iafrate AJ, Ladanyi M, Kris MG, Johnson BE, Bunn PA, Minna JD (2015). Multi-institutional Oncogenic Driver Mutation Analysis in Lung Adenocarcinoma: The Lung Cancer Mutation Consortium Experience. Journal of Thoracic Oncology.

[41] Barreto AM, Schwartz GG, Woodruff R, Cramer SD (2000). 25-Hydroxyvitamin D3, the prohormone of 1,25-dihydroxyvitamin D3, inhibits the proliferation of primary prostatic epithelial cells. Cancer Epidemiology, Biomarkers & Prevention.

[42] Schwartz GG, Eads D, Rao A, Cramer SD, Willingham MC, Chen TC, Jamieson DP, Wang L, Burnstein KL, Holick MF, Koumenis C (2004). Pancreatic cancer cells express 25-hydroxyvitamin D-1 alpha-hydroxylase and their proliferation is inhibited by the prohormone 25-hydroxyvitamin D3. Carcinogenesis.

[43] Munetsuna E, Kawanami R, Nishikawa M, Ikeda S, Nakabayashi S, Yasuda K, Ohta M, Kamakura M, Ikushiro S, Sakaki T (2014). Anti-proliferative activity of 25-hydroxyvitamin D3 in human prostate cells. Molecular and Cellular Endocrinology.

[44] Hummel DM, Thiem U, Hobaus J, Mesteri I, Gober L, Stremnitzer C, Graca J, Obermayer-Pietsch B, Kallay E (2013). Prevention of preneoplastic lesions by dietary vitamin D in a mouse model of colorectal carcinogenesis. The Journal of Steroid Biochemistry and Molecular Biology.

[45] Chiang KC, Yeh CN, Lin KJ, Su LJ, Yen TC, Pang JH, Kittaka A, Sun CC, Chen MF, Jan YY, Chen TC, Juang HH, Yeh TS (2014). Chemopreventive and chemotherapeutic effect of dietary supplementation of vitamin D on cholangiocarcinoma in a Chemical-Induced animal model. Oncotarget.

[46] Meeker S, Seamons A, Paik J, Treuting PM, Brabb T, Grady WM, Maggio-Price L (2014). Increased dietary vitamin D suppresses MAPK signaling, colitis, and colon cancer. Cancer Res.

[47] Jeong Y, Swami S, Krishnan AV, Williams JD, Martin S, Horst RL, Albertelli MA, Feldman BJ, Feldman D, Diehn M (2015). Inhibition of Mouse Breast Tumor-Initiating Cells by Calcitriol and Dietary Vitamin D. Molecular Cancer Therapeutics.

[48] Rossdeutscher L, Li J, Luco AL, Fadhil I, Ochietti B, Camirand A, Huang DC, Reinhardt TA, Muller W, Kremer R (2015). Chemoprevention activity of 25-hydroxyvitamin D in the MMTV-PyMT mouse model of breast cancer. Cancer Prevention Research.

[49] Mazzilli SA, Hershberger PA, Reid ME, Bogner PN, Atwood K, Trump DL, Johnson CS (2015). Vitamin D Repletion Reduces the Progression of Premalignant Squamous Lesions in the NTCU Lung Squamous Cell Carcinoma Mouse Model. Cancer Prevention Research.

[50] Swami S, Krishnan AV, Wang JY, Jensen K, Horst R, Albertelli MA, Feldman D (2012). Dietary vitamin D(3) and 1,25-dihydroxyvitamin D(3) (calcitriol) exhibit equivalent anticancer activity in mouse xenograft models of breast and prostate cancer. Endocrinology.

[51] Matilainen JM, Malinen M, Turunen MM, Carlberg C, Vaisanen S (2010). The number of vitamin D receptor binding sites defines the different vitamin D responsiveness of the CYP24 gene in malignant and normal mammary cells. The Journal of Biological Chemistry.

[52] Seuter S, Pehkonen P, Heikkinen S, Carlberg C (2013). Dynamics of 1alpha,25-dihydroxyvitamin D3-dependent chromatin accessibility of early vitamin D receptor target genes. Biochimica et Biophysica Acta.

[53] Radermacher J, Diesel B, Seifert M, Tilgen W, Reichrath J, Fischer U, Meese E (2006). Expression analysis of CYP27B1 in tumor biopsies and cell cultures. Anticancer Research.

[54] Kong J, Xu F, Qu J, Wang Y, Gao M, Yu H, Qian B (2015). Genetic polymorphisms in the vitamin D pathway in relation to lung cancer risk and survival. Oncotarget.

[55] Dardenne O, Prud'homme J, Arabian A, Glorieux FH, St-Arnaud R (2001). Targeted inactivation of the 25-hydroxyvitamin D(3)-1(alpha)-hydroxylase gene (CYP27B1) creates an animal model of pseudovitamin D-deficiency rickets. Endocrinology.

[56] Panda DK, Miao D, Tremblay ML, Sirois J, Farookhi R, Hendy GN, Goltzman D (2001). Targeted ablation of the 25-hydroxyvitamin D 1alpha-hydroxylase enzyme: evidence for skeletal, reproductive, and immune dysfunction. Proceedings of the National Academy of Sciences of the United States of America.

[57] Vanhooke JL, Prahl JM, Kimmel-Jehan C, Mendelsohn M, Danielson EW, Healy KD, DeLuca HF (2006). CYP27B1 null mice with LacZreporter gene display no 25-hydroxyvitamin D3–1alpha-hydroxylase promoter activity in the skin. Proceedings of the National Academy of Sciences of the United States of America.

[58] Upadhyay SK, Verone A, Shoemaker S, Qin M, Liu S, Campbell M, Hershberger PA (2013). 1,25-Dihydroxyvitamin D3 (1,25(OH)2D3) Signaling Capacity and the Epithelial-Mesenchymal Transition in Non-Small Cell Lung Cancer (NSCLC): Implications for Use of 1,25(OH)2D3 in NSCLC Treatment. Cancers.

[59] Yao Z, Fenoglio S, Gao DC, Camiolo M, Stiles B, Lindsted T, Schlederer M, Johns C, Altorki N, Mittal V, Kenner L, Sordella R (2010). TGF-beta IL-6 axis mediates selective and adaptive mechanisms of resistance to molecular targeted therapy in lung cancer. Proceedings of the National Academy of Sciences of the United States of America.

[60] Sequist LV, Waltman BA, Dias-Santagata D, Digumarthy S, Turke AB, Fidias P, Bergethon K, Shaw AT, Gettinger S, Cosper AK, Akhavanfard S, Heist RS, Temel J, Christensen JG, Wain JC, Lynch TJ (2011). Genotypic and histological evolution of lung cancers acquiring resistance to EGFR inhibitors. Science Translational Medicine.

[61] Byers LA, Diao L, Wang J, Saintigny P, Girard L, Peyton M, Shen L, Fan Y, Giri U, Tumula PK, Nilsson MB, Gudikote J, Tran H, Cardnell RJ, Bearss DJ, Warner SL (2013). An epithelial-mesenchymal transition gene signature predicts resistance to EGFR and PI3K inhibitors and identifies Axl as a therapeutic target for overcoming EGFR inhibitor resistance. Clinical Cancer Research.

[62] Zhang Q, Kanterewicz B, Buch S, Petkovich M, Parise R, Beumer J, Lin Y, Diergaarde B, Hershberger PA (2012). CYP24 inhibition preserves 1alpha,25-dihydroxyvitamin D(3) anti-proliferative signaling in lung cancer cells. Molecular and Cellular Endocrinology.

[63] Polson C, Sarkar P, Incledon B, Raguvaran V, Grant R (2003). Optimization of protein precipitation based upon effectiveness of protein removal and ionization effect in liquid chromatography-tandem mass spectrometry. Journal of Chromatography B, Analytical technologies in the biomedical and life sciences.

[64] Ding S, Schoenmakers I, Jones K, Koulman A, Prentice A, Volmer DA (2010). Quantitative determination of vitamin D metabolites in plasma using UHPLC-MS/MS. Analytical and Bioanalytical Chemistry.

[65] Menezes RJ, Cheney RT, Husain A, Tretiakova M, Loewen G, Johnson CS, Jayaprakash V, Moysich KB, Salgia R, Reid ME (2008). Vitamin D receptor expression in normal, premalignant, and malignant human lung tissue. Cancer Epidemiology, Biomarkers & Prevention.

