# Supplementary material for: Diet-derived 25-hydroxyvitamin D3 activates vitamin D receptor target gene expression and suppresses EGFR mutant non-small cell lung cancer growth in vitro and in vivo
Source: Oncotarget. 2015 Dec 8;7(1):995–1013. doi: 10.18632/oncotarget.6493 (PMC4808047; doi:10.18632/oncotarget.6493)
Supplement: Supplementary file 1 [file oncotarget-07-0995-s001.pdf]

SUPPLEMENTARY FIGURES

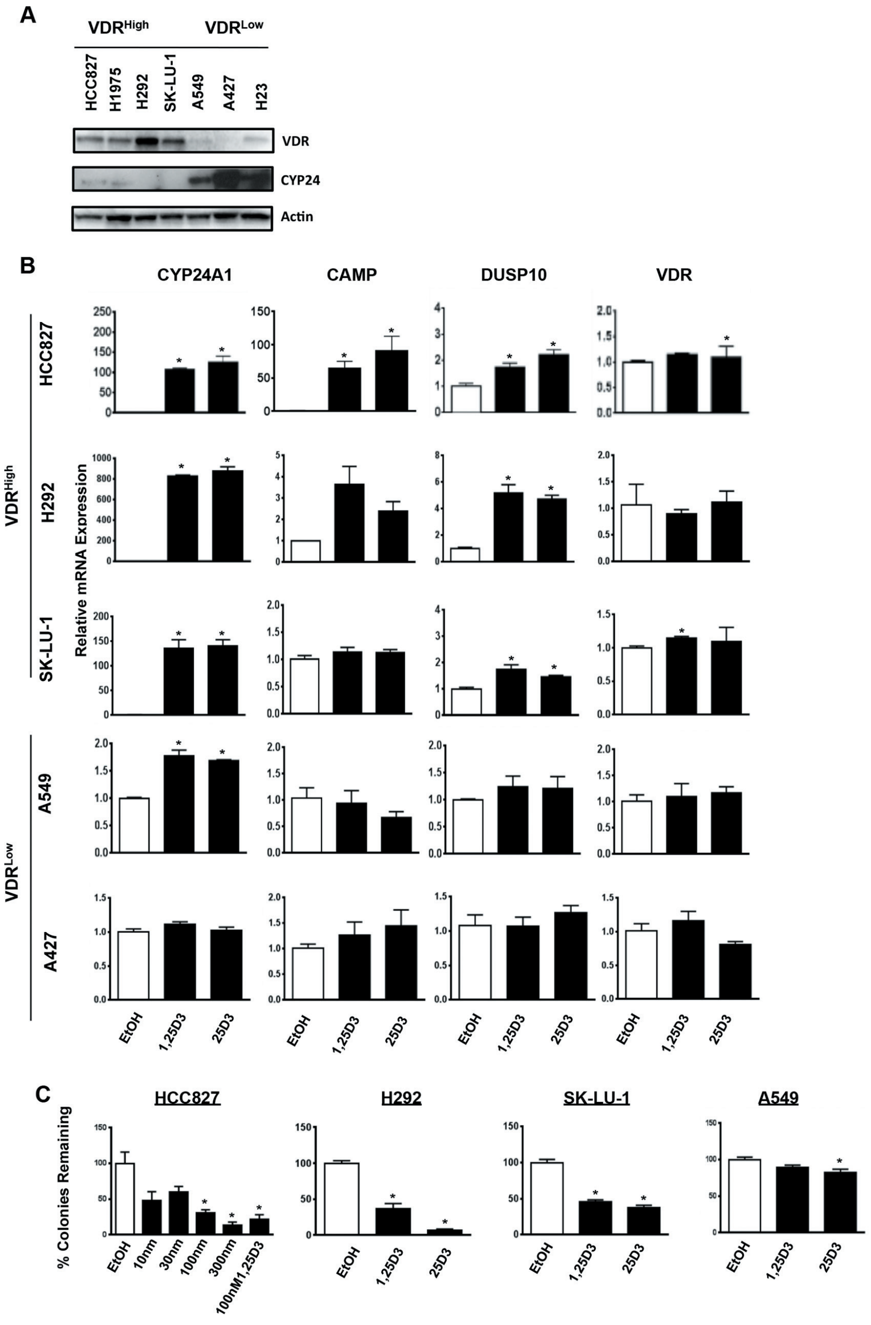

Supplementary Figure S1: VDRhigh NSCLC cells respond to 25D3 treatment. A. A panel of NSCLC cells with varying VDR protein expression was examined for 25D3 responsiveness. HCC827, H292, H1975, and SK-LU-1 are considered VDRhigh cell lines due to the higher basal VDR protein expression. The cell lines A549, A427 and H23 are considered VDRlow and express very low basal VDR, and higher CYP24A1 protein. **B.** VDRhigh and VDRlow cells were treated with vehicle control, 100nm 1,25D3 or 1uM 25D3 for six, 24 or 48 hours (only six hour data shown). VDRhigh cells display significant induction of VDR target genes when exposed to both metabolites, while VDRlow cells do not respond to the same magnitude. **C.** Furthermore, VDRhigh cells are at least 50% growth inhibited when treated with vitamin D3 metabolites, including various doses of 25D3 (HCC827). VDRlow cells only reach a maximal of 11% growth inhibition upon 25D3 exposure. Asterisks represent $p < 0.05$ by Student's *t* test and are a comparison between the ethanol treated group and either the 1,25D3 or 25D3 treatment.

Cell line mRNA data

Cell Line	Av. Corrected Ct	Fold Change
H292	11.2	10.3
A549	11.7	7.18
H3122	12.8	3.35
HCC827	14.1	0.99

COSMIC Database

Copy Number Gain	23%
Copy Number Loss	12.4%

Supplementary Figure S2: *CYP27B1* mRNA expression is variable in NSCLC. *CYP27B1* mRNA expression was surveyed across NSCLC cell lines (left) and the COSMIC database. RNA was isolated from cell lines and qRT-PCR was performed to examine *CYP27B1* expression. Shown here are the raw Ct values with GAPDH subtracted (average corrected Ct). Data from the COSMIC database further indicate that the *CYP27B1* gene is subjected to copy number gains or losses.
